# PTOLEMI: Personalized Cancer Treatment through Machine Learning-Enabled Image Analysis of Microfluidic Assays

**DOI:** 10.3390/diagnostics13193075

**Published:** 2023-09-28

**Authors:** Bernard Moerdler, Matan Krasner, Elazar Orenbuch, Avi Grad, Benjamin Friedman, Eliezer Graber, Efrat Barbiro-Michaely, Doron Gerber

**Affiliations:** Life Sciences Faculty and Nanotechnology Institute, Bar-Ilan University, Ramat Gan 5290002, Israel

**Keywords:** machine learning, image processing, point of care diagnostics, microfluidics

## Abstract

Contemporary personalized cancer diagnostic approaches encounter multiple challenges. The presence of cellular and molecular heterogeneity in patient samples introduces complexities to analysis protocols. Conventional analyses are manual, reliant on expert personnel, time-intensive, and financially burdensome. The copious data amassed for subsequent analysis strains the system, obstructing real-time diagnostics at the “point of care” and impeding prompt intervention. This study introduces PTOLEMI: Python-based Tensor Oncological Locator Examining Microfluidic Instruments. PTOLEMI stands out as a specialized system designed for high-throughput image analysis, particularly in the realm of microfluidic assays. Utilizing a blend of machine learning algorithms, PTOLEMI can process large datasets rapidly and with high accuracy, making it feasible for point-of-care diagnostics. Furthermore, its advanced analytics capabilities facilitate a more granular understanding of cellular dynamics, thereby allowing for more targeted and effective treatment options. Leveraging cutting-edge AI algorithms, PTOLEMI rapidly and accurately discriminates between cell viability and distinct cell types within biopsy samples. The diagnostic process becomes automated, swift, precise, and resource-efficient, rendering it well-suited for point-of-care requisites. By employing PTOLEMI alongside a microfluidic cell culture chip, physicians can attain personalized diagnostic and therapeutic insights. This paper elucidates the evolution of PTOLEMI and showcases its prowess in analyzing cancer patient samples within a microfluidic apparatus. While the integration of machine learning tools into biomedical domains is undoubtedly in progress, this study’s innovation lies in the fusion of PTOLEMI with a microfluidic platform—an integrated, rapid, and independent framework for personalized drug screening-based clinical decision-making.

## 1. Introduction

Over the past half-century, cancer has emerged as a formidable global health challenge, ranking as the second leading cause of death worldwide. According to the World Health Organization, in 2022 alone, an estimated 10 million cancer-related deaths occurred [1], highlighting the urgent need for improved diagnostic and treatment approaches. Despite significant advancements in medical science, the complexity and heterogeneity of cancer pose major obstacles in achieving optimal treatment outcomes. The one-size-fits-all approach, where patients with the same tumor type and genetic profile receive identical treatment protocols, fails to account for the varied responses and individual differences in drug efficacy [2].

Personalized medicine has emerged as a promising paradigm in cancer treatment, aiming to tailor therapies to the specific characteristics and needs of each patient [3,4]. However, the implementation of personalized medicine faces significant challenges, particularly in terms of accurate diagnosis and expedient, high-throughput analysis. Currently, diagnostic methods involve profiling a tumor’s DNA, RNA, and protein composition, followed by chemosensitivity and resistance assays conducted using various disease models. While these methods have shown some improvement in clinical outcomes, they are limited by the inherent cellular and molecular heterogeneity within tumors [5]. This heterogeneity makes it difficult to capture the full spectrum of tumor behavior and response to treatment. Moreover, these methods are time-consuming and costly, which poses a serious problem for timely interventions. Studies have demonstrated that delayed treatment initiation increases the risk of disease progression and mortality. For instance, the Mayo Clinic reports a five-year survival rate of 60% for individuals diagnosed with early stage lung cancer, whereas those diagnosed with late-stage lung cancer that have metastasized to other parts of the body have a survival rate of only 6% [6]. This highlights the urgency in expediting the diagnostic and analysis processes.

To tackle these challenges, several alternatives have been proposed, the most promising of which is the integration of a cytotoxicity and resistance assay (CSRA) model within a microfluidic platform [7]. Microfluidics, a field which has been around since the 1990′s and has increased in popularity ever since, is the science of manipulating tiny volumes of fluids ranging from picolitres to microliters within channels ranging in size from one micron to one millimeter [8]. This dynamic interdisciplinary field is now commonly incorporated into a wide variety of regularly used molecular biology techniques [9], although applying microfluidics in analysis processes of patient samples such as tumor or tissue biopsies is rare. There is no clinically approved solution at the point of care. We previously presented an experimental CSRA-based platform [10]. This platform has the potential to provide clinicians with important information about cell response to various drugs at different concentrations or combinations.

This CSRA platform faces a major bottleneck in the analysis of the massive amount of data generated for each patient before becoming relevant as a point of care solution. The data come from biopsy samples that contain a diverse mix of cell types, such as fibroblasts, immune cells, red blood cells, and others. The cell types vary depending on the tissue the sample was taken from, adding more complexity to the analysis. In addition, the amount of data from a single patient also adds to this bottleneck. To illustrate this problem, consider a typical scenario where 10 drugs are tested at 3 concentrations. This results in a total of 3072 image per patient. Assuming that a technician can analyze 500 image points per day, it would take a week to analyze the whole dataset for just one patient. This shows the critical gap between the urgency of personalized treatment and the slowness of current analysis methods. Therefore, there is a pressing need for innovative, efficient, and cost-effective solutions for automating the analysis.

Over the last decade, researchers have turned to machine learning and image analysis techniques to expedite the data analysis required for proper patient diagnosis. By harnessing the power of artificial intelligence (AI) and, more specifically, deep learning algorithms, they aim to automate the detection and analysis of cancer cells in microscope images. The automation of said processes enables the identification and quantification of both live and dead cancer cells, providing crucial information for determining the most optimal course of treatment [11,12]. Ultimately, the integration of machine learning and image analysis in biomedical applications as a whole and in point of care oncological diagnostic devices in particular holds great promise for advancing personalized medicine and revolutionizing cancer treatment strategies.

Conventional image analysis software like ImageJ, NIS Elements, ZEN Microscopy Software by ZEISS, even in their latest versions, do not provide a sufficient solution for clinical point of care applications. These software packages rely on pixel thresholding to distinguish between cellular entities and the background. However, in the cases of cells within polydimethylsiloxane (PDMS)-made chambers, this threshold becomes somewhat blurry. This predicament leads to a fundamental impediment in automating image analysis, primarily arising from the misclassification of the PDMS chamber “walls”. This leads to inaccuracies that require manual corrections. Thus, such a solution is not relevant for a point of care setting, which requires full automation.

In line with these objectives, PTOLEMI, the “Python-based Tensor Oncological Locator Examining Microfluidic Instruments”, has been developed as an advanced platform for analyzing microscope images in the context of cancer cell detection within PDMS microfluidic devices. PTOLEMI leverages state-of-the-art AI algorithms to rapidly and accurately distinguish different cell types, including various cancer types, as well as non-cancerous cells such as fibroblasts, red blood cells, and immune cells, from biopsy samples. This capability enables comprehensive analysis of cellular composition, enhancing the accuracy and specificity of personalized cancer treatment plans. PTOLEMI’s algorithm continuously learns from the data generated by microfluidic experiments, allowing it to improve over time. The combination of PTOLEMI’s image analysis with the CSRA-based microfluidic assay will lead to more robust and informed decision-making in personalized cancer treatment.

## 2. Methods

### 2.1. Chip Manufacturing

Microfluidic devices are produced by soft-lithography processes from poly-di-methyl siloxane (PDMS) [13], which is transparent, permeable to gas, and bio-compatible. Our microfluidic devices incorporated pneumatic valves that controlled the reagent flow within the device (“integrated microfluidics”). The dimensions of microfluidic devices were in the scale of microns, and the reagents’ volumes were of numerous microliters and thus can run high-throughput experiments [14]. PDMS devices were manufactured using standard methods in Prof. Gerber’s lab. Briefly, the flow (cell culturing) and control (valves) layers were prepared separately on silicone molds casting silicone elastomer polydimethylsiloxane (PDMS, SYLGARD 184, Dow Corning, Midland, MI, USA). For the production of the control layer, PDMS and curing agent are mixed at a 5:1 ratio, followed by degassing for 15 min, baking for an hour, and access-hole-piercing steps. The flow layer was prepared similarly, except for the application of a 20:1 ratio of PDMS to curing agent. The flow layer contained two different heights, in which different components were located. The “forks filters” that delivered medium to the cultivated cells were located at the lower area of the flow layer, whereas the chambers and other tubes were located at the upper zone of the layer to allow for optimal culture media diffusion without the risk of cross contamination or cell escape. After the preparation of both layers, they were aligned using a home-made semi-automatic alignment system. The chip was then placed in an oven at 80C for full curing. Holes were punched to allow the connection of tubing via pins and the flowing of air or fluids into the chip during the experiment. 

### 2.2. Cell Cultivation

H-1650 EGFR cell line was purchased from American Type Culture Collection (ATCC) Rockville, MD, USA and cultivated in 10 cm surface treated Petri Dish (Lumitron, Kiryat Arieh, Petah Tikva, Israel). Cells were suspended in RPMI 1640 medium, supplemented with 10% FBS (BI, BEIT HAEMEK LTD, Israel), 1% penicillin + streptomycin (BI, BEIT HAEMEK LTD, Israel), and 1% L-glutamine (BI, BEIT HAEMEK LTD, Israel). The cells were incubated in a CO_2_ control incubator (New Brunswick Galaxy 170 S) at 37 °C, 5% CO_2_. A maximum of 15 passages were applied for each tissue culture dish.

### 2.3. Chip Operation

The flow of medium/cells was regulated by using a proprietary pneumatic system and a computational controller (regulated semi-automatically). Working pressure for cell flow was 2–3 PSI. Input and output control valves were operated at 20 PSI. The temperature, humidity, and CO_2_ were controlled by the fluorescent microscope incubator built-in system (Bold Line, Okolab, Rovereto, TN, Italy).

### 2.4. Microfluidic Experimental Assay

For surface treatment, the chip channels were washed with ethanol (20 min), PBS (15min), and poly-lysine (20 min) to assist cell adhesion (where needed), and Pluronic F-127 (5%) (20 min) for blocking in regions where cells were unwanted. Cells were then introduced into the main channel and pushed into the incubation chambers by horizontal flow. The neck valve was then closed, the main flow channels were thoroughly washed with trypsin (0.25% BI, Israel) to remove any residual cells, which, in turn, was washed out with PBS (15 min), and, finally, replaced with RPMI 1640. The cells were then left for 1–2 h in the cultivation chamber, with no flow (preventing any shear forces) to allow for adhesion and acclimation. Following this acclimation period, cultivation media flow began, and the cells were left for another 24–48 h (depending on experimental protocol), in which they were imaged every 2 h as needed.

### 2.5. Cell Viability Assessment

Custom assays for cell viability evaluation were applied. Dead cells were stained with propidium iodide (PI) (Sigma, Rehovot, Israel) (30 µL PI in 15 mL RPMI medium). All cells’ nuclei (live and dead) were stained by Hoechst 33342 stain (bis-benzamide H33342 trihydrochloride, Sigma, Rehovot, Israel) (30 µL in 15 mL RPMI medium). A solution of the two dyes was prepared and flowed into the chip. At the beginning of the experiment, the dye solution was applied. Once the flow stopped, the cells were incubated with the dyes for 2 h at 37 °C 5% CO_2_ to allow for cell staining. Once the acclimation period ended, the cells were continuously administered the solution for the duration of the experiment (24 h for acclimation and 24–48 h treatment) at a pressure of 2–3 PSI.

### 2.6. Chip Imaging and Manual Data Analysis

Imaging was performed by Nikon Eclipse Ti. Images were acquired via NIS Elements software (ver. 4.20.01 Nikon, NY, USA) and processed with NIS Elements Analysis software (ver. 40.20.01 Nikon, NY, USA). To allow automatic filming of specific chambers, special attention was given to linear alignment of the entire chip to the slide borders during the manufacturing procedures. Automatic analysis was complemented by manual curation due to visual interference from the microfluidic device.

### 2.7. AI Model Description

The AI model utilized in this study was a Faster Region-based Convolutional Neural Network (Faster R-CNN) architecture, which integrated a ResNet backbone network. Faster R-CNN is well-known for its exceptional object detection capabilities and proves to be an ideal choice for accurate cell analysis in microscope images.

The Faster R-CNN model consists of two key components: the ResNet backbone network and the Region Proposal Network (RPN). The ResNet backbone network is responsible for extracting essential features from the input microscope images. Its deep convolutional layers with skip connections effectively address the vanishing gradient problem, enabling the training of deeper networks and enhancing the model’s ability to discern intricate cellular patterns and features.

The Region Proposal Network (RPN) operates on the convolutional feature map generated by the ResNet backbone. It efficiently proposes candidate regions containing cells by systematically analyzing the likelihood of each anchor box encompassing a cell. The RPN identifies regions with high confidence scores, which are then subjected to detailed analysis by the Fast R-CNN detector.

Within the Faster R-CNN architecture, the Fast R-CNN detector plays a pivotal role in precise cell classification and localization within the proposed candidate regions. Leveraging distinctive cellular features, the Fast R-CNN accurately determines the cytotoxicity or resistance of cells to various treatments.

In PTOLEMI, we adopted the Faster R-CNN architecture as the foundational framework for object detection and classification, bringing together a series of intricately designed components. The model initiated with a backbone Convolutional Neural Network (CNN), typically a deep network like ResNet or VGG. This CNN transformed the input image into a high-dimensional feature map through a sequence of convolutional, batch normalization, and ReLU activation layers [15]. The resulting feature map served as a nuanced detector, capturing various image attributes such as texture and morphology. Subsequently, a Region Proposal Network (RPN) scanned this feature map using a sliding window approach to identify potential object-containing regions. These proposed regions were then refined through a Region of Interest (RoI) Align layer, which employed bilinear interpolation to extract fixed-size feature vectors while preserving spatial information. [16]. Finally, these feature vectors were processed by two fully connected layers for classification and bounding box regression. The classification layer utilized a softmax function to calculate class probabilities, whereas the regression layer refined the bounding box coordinates to accurately encapsulate the object [17]. This meticulous architecture enabled Faster R-CNN to effectively extract features for precise object detection and classification, a capability that is leveraged in PTOLEMI for cellular analysis in microfluidic assays.

### 2.8. Validation and Testing

To ensure the AI model’s reliability and robustness, rigorous validation and testing protocols were meticulously executed. The dataset was judiciously partitioned into training and validation sets, adopting a 70:30 ratio respectively, ensuring ample data for both training and evaluation phases.

To further assess the model’s generalization capabilities and minimize overfitting, the study embraced k-fold cross-validation. By partitioning the dataset into k subsets and iteratively training and validating on different combinations, the AI model’s accuracy and consistency were thoroughly examined. In PTOLEMI, we employed Faster R-CNN as the foundational architecture for object detection and classification, bringing together a series of intricately designed components. The model initiated with a backbone Convolutional Neural Network (CNN), commonly a deep network like ResNet or VGG, which transformed the input image into a high-dimensional feature map through a sequence of convolutional, batch normalization, and ReLU activation layers [15]. This feature map serves as a nuanced detector, capturing a variety of image attributes, such as texture and morphology-nuanced detector. Following the generation of this feature map, a Region Proposal Network (RPN) scanned it using a sliding window approach to identify potential object-containing regions [17]. These proposed regions were then refined through a Region of Interest (RoI) Align layer, which employed bilinear interpolation to extract fixed-size feature vectors while preserving spatial information [16]. Finally, these vectors were fed into two fully connected layers for classification and bounding box regression. The classification layer employed a softmax function to calculate class probabilities, whereas the regression layer refined the bounding box coordinates to accurately encapsulate the object [17]. Through this meticulous architecture, faster R-CNN effectively extracted features for precise object detection and classification—a capability that is exploited in PTOLEMI for cellular analysis in microfluidic assays.

### 2.9. Implementation Details

The AI-based cell analysis system was developed using Python as the programming language, along with several essential libraries for building and training the deep learning model. Below are the key libraries used in the system:

Python: Python was the primary programming language used for building the AI-based cell analysis system. Its simplicity and extensive libraries make it an excellent choice for handling complex AI tasks.

PyTorch: The core of the AI model was built using PyTorch, a popular open-source deep learning framework. PyTorch provided efficient tensor operations, automatic differentiation, and GPU acceleration, making it suitable for creating and training complex neural networks.

Torchvision: The torchvision library is an integral part of PyTorch, providing various datasets, pre-trained models, and image transformation utilities that facilitate training object detection models.

Pandas: The pandas library was used for data manipulation and handling tabular data. In this case, it was used to read and preprocess the label data, either from XML files or CSV files, to create a dataset for training.

Tqdm: The tqdm library was used to display progress bars during training and validation steps, providing a visual indication of the training progress.

NumPy: NumPy was employed for efficient numerical computations and data handling. It was used for processing image data and working with multidimensional arrays.

Os: The os module is a built-in Python library used for interacting with the operating system. It was employed in the implementation to manage file paths and handle directory operations.

Random: The random module provided functions for generating random numbers, which may be used for data augmentation or shuffling the dataset during training.

These libraries collectively formed the foundation of the AI-based cell analysis system, enabling efficient data handling, training, and inference on cell images, resulting in accurate and reliable cell detection and classification.

## 3. Results

**Figure 1:**
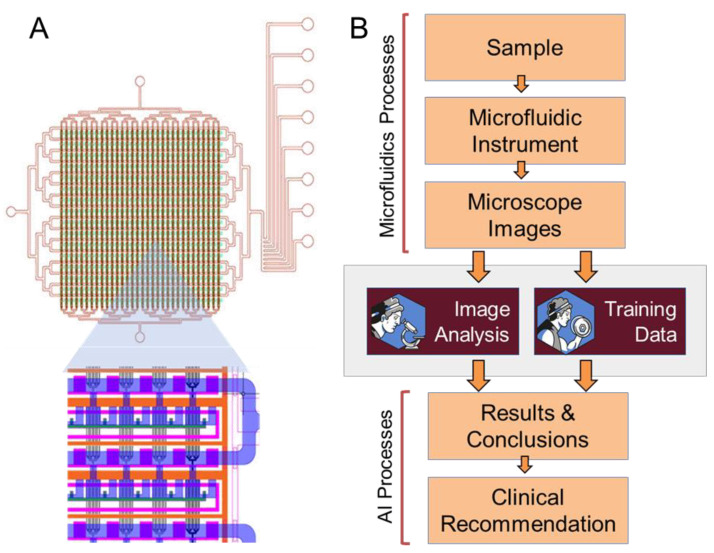
Cell culture microfluidic device schematic and workflow. (**A**) The cell culture chip (upper) includes 16 × 16 chambers (at the size of 350 × 275 µm at 12–15 µm height) for cell cultivation and a set of valves controlling reagent flow. Zooming in (lower) presents the neck and filter valves (green and orange, respectively) that prevent cross-contamination between channels and chamber while enabling culture media (flowing at 2–3 psi) to diffuse in and out of the chambers freely. (**B**) Application use case overview in a clinical setting. Patient samples are tested against a panel of possible therapies and analyzed by PTOLEMI to provide evidence for sensitivity or resistance. Samples are simultaneously used to train the AI and to further improve accuracy in future sample analysis.

In the concurrent study, a biopsy was taken from a cancer patient and seeded inside of a microfluidic device fabricated for cell cultivation and subsequent analysis. The chip comprised 256 different cultivation chambers organized into a 16 × 16 array for testing against a predetermined drug panel depending on the type of cancer in question (Figure 1A). The cells were cultivated in the chip for 36–48 h, during which they were continuously exposed to a cultivation medium that included a drug treatment along with fluorescent dyes via diffusion through microscopic microfluidic channels that mimicked venous/arterial diffusion in the body (Figures S1 and S2). Throughout the drug exposure period, the cells were imaged at predetermined time intervals (typically every 2 h) via fluorescent microscopy, and the resulting images were analyzed to determine the cytotoxicity or resistance of the cells to each treatment, respectively (Figure 1B) [17].

Just a single one of these assays resulted in 256 cultivation chamber images across 25 time points for a grand total of 6400 images that required analysis. Assuming a seasoned technician could manually analyze an image (i.e., count the total number of cells and categorize them into live vs. dead) in approximately one minute, this would require 106 h and 40 min (nearly 3 standard work weeks) of analysis for a single patient. Thus, developing such diagnostics for the point of care is challenging. For a personalized approach to be scalable and point of care ready, technological tools such as PTOLEMI are crucial to sift through this data in a high-throughput, automated, accurate, and eventually in an on-the-fly manner.

PTOLEMI achieves this via a two-pronged approach. For starters, conservatively calculating (25 s for 150 images) and analyzing the 6400 images from each assay would take a little less than 18 min to run (17:47), representing an over 360-fold conservation in time or 99.72%. In addition to the increased speed, each time the program runs, it uses data from the image analysis to both analyze the image to assist the physician in reaching a diagnostic or therapeutic conclusion as well as using the data to train itself, thereby further increasing the accuracy of its database by incorporating new images into it. (Figure 1B). 

PTOLEMI has a structure of neurons known as an artificial neural network (ANN) that it employs to analyze the images. The ANN has multiple layers of neurons that communicate with each other through weights and biases, which control how the data are transferred from one layer to the next. The ANN is capable of processing complex data and identifying relevant features from the images (Figure S3). The ANN adapts to the labeled data by modifying its weights and biases through a process known as backpropagation. This process minimizes the error between the expected and actual outputs of the ANN and enhances its accuracy and efficiency over time. The ANN can detect the patterns and features that signify whether a cell is alive or dead based on its fluorescence, intensity, and morphology.

**Figure 2:**
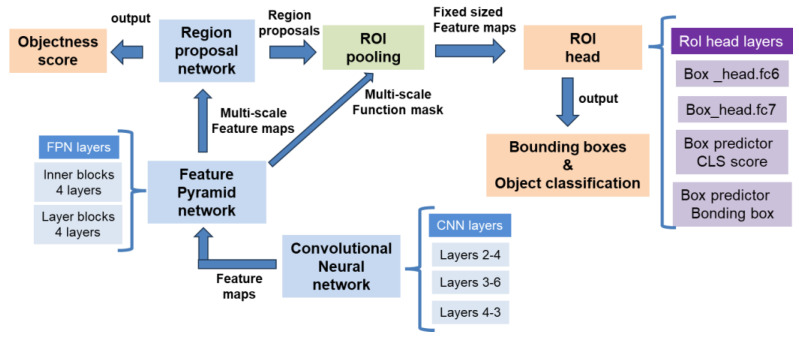
PTOLEMI’s architecture overview. Composition and interactions of PTOLEMI’s Faster R-CNN backbone, a crucial component for object detection. It consists of a Convolutional Neural Network (CNN) as the feature extractor, a Feature Pyramid Network (FPN) for handling objects of different sizes, and a Region Proposal Network (RPN) to generate candidate bounding boxes. The RoI Pooling module connects the FPN and RPN outputs to produce fixed-sized feature maps. Finally, the RoI Head performs object classification and bounding box regression. This multi-layered architecture enables accurate and efficient object detection from input images.

Transitioning to PTOLEMIs Faster R-CNN backbone, which comprises several key components, each consisting of multiple layers that play a crucial role in the object detection process. At its foundation, the Convolutional Neural Network (CNN) extracts essential features from the input image, allowing the model to comprehend visual patterns and content. The Feature Pyramid Network (FPN) builds upon the CNN’s output by creating a feature pyramid, ensuring effective handling of objects of different sizes. Another critical element is the Region Proposal Network (RPN), which is responsible for generating region proposals—candidate bounding boxes likely to contain objects. The RPN utilizes specific layers to predict the positions and objectness scores of the proposals, guiding the model’s attention to potential object locations. The Region of Interest (RoI) Pooling module connects to the output of both the FPN and the RPN. It leverages selected features from the FPN and the RoI proposals from the RPN to generate fixed-sized feature maps. This transformation ensures consistent-size representations for each proposal, facilitating further processing. Finally, the RoI Head constitutes the last stage of the backbone. It employs specific layers to perform two essential tasks: object classification and bounding box regression. The RoI Head predicts the probabilities of different object categories and refines the coordinates of the bounding boxes, leading to precise object classification and localization (Figure 2). 

This integration of the ANN and the Faster R-CNN backbone forms the foundation of PTOLEMI’s exceptional performance in cell analysis and object detection. By leveraging the ANN’s ability to process complex image data and detect meaningful patterns, PTOLEMI gains a deep understanding of the cell images, enabling it to make accurate decisions on cell viability. The Faster R-CNN backbone, with its feature extraction and region proposal mechanisms, ensures precise and efficient object detection, crucial for identifying and localizing cells of interest [17]. This combined power allows PTOLEMI to effectively handle large-scale image datasets with diverse cellular structures and varying imaging conditions. Moreover, the adaptive nature of the ANN, continuously refining its weights and biases through backpropagation, enables PTOLEMI to adapt to new datasets and enhance its accuracy over time. 

**Figure 3:**
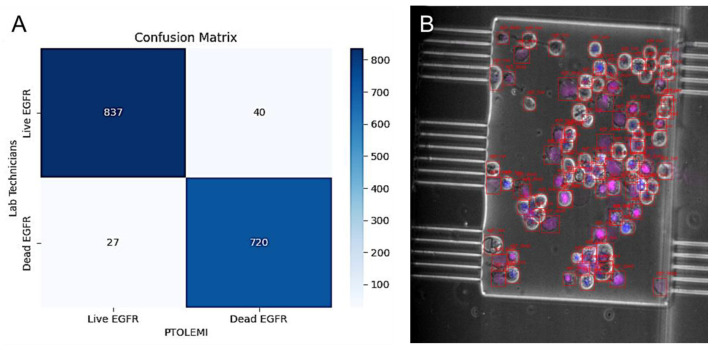
Viability assessment of EGFR cell line—the comparison between human vs. PTOLEMI’s AI assessments. (**A**) Confusion Matrix for PTOLEMI’s Classification Results on samples of EGFR cell line (150 Images): The confusion matrix shows True Positive (TP), False Negative (FN), False Positive (FP), and True Negative (TN) values, indicating PTOLEMI’s performance on the image dataset. (**B**) Visual representation of image identification: H-1650 EGFR cells were seeded in the microfluidic device and stained with Propidium Iodide (magenta, dead cells) and Hoechst 33342 (Blue, live). Image analysis was then conducted using PTOLEMI.

In order to showcase the accuracy and speed, PTOLEMI was used on a dataset of 150 images of H-1650 cells that contained a mutation in their Epidermal Growth Factor Receptor gene (EGFR). The confusion matrix (Figure 3) indicated the results and showcased that PTOLEMI could precisely classify the cells as either live or dead. PTOLEMI accurately identified 837 live cells (true positives) and only misclassified 27 dead cells as live (false positives). PTOLEMI also accurately identified 720 dead cells (true negatives) and only misclassified 40 live cells as dead (false negatives). These results could be used to calculate an accuracy of nearly 96%. An additional dataset of images from Glioma stem cells imaged under the same conditions was also analyzed using PTOLEMI with similar results (Figure S4) [18].

**Figure 4:**
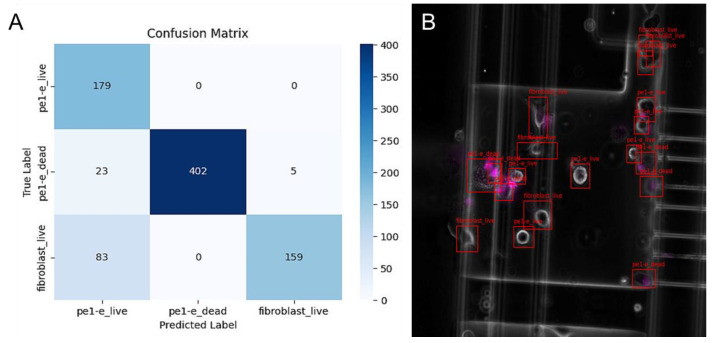
Viability assessment of patient pleural effusion cells (PE1-E) within a mixed cellular environment—the comparison between human vs. PTOLEMI’s AI assessments. (**A**) Confusion Matrix for PTOLEMI’s Classification; The confusion matrix was applied on PE1-E sample, which included cancer cells and fibroblasts (122 Images): The confusion matrix shows the classification of cells comparing predicted vs. true labels. (**B**) Visual representation of image identification: PE1-E cells were seeded in the microfluidic device and stained with propidium iodide (magenta, dead cells); image analysis was then conducted using PTOLEMI. The sample includes cancer cells as well as fibroblasts. To differentiate the cells by morphology rather than the standard pixel thresholding prevalent in other models, fibroblast-specific fluorescent markers were not used to train PTOLEMI.

Having determined the system binary classification capabilities of live vs. dead cells for the cell lines sample, which was a homogenic sample of one type of cell, PTOLEMI was challenged even more by testing its capabilities on primary patient samples that were heterogeneously regarding the types of cells these samples contained (Figure 4). This important step aimed towards clinical point-of-care conditions.

Remarkably, PTOLEMI demonstrated its ability to accurately differentiate between the different cell types in this complex sample. Out of the living PE1-E cells, 179 live cells were correctly identified, whereas 23 dead cells were misidentified as live cells, and 83 fibroblast cells were also mistakenly identified as live PE1-E cells.

When it came to the dead PE1-E cells, PTOLEMI identified 402 of them correctly and did not misidentify any of them as live cells. Furthermore, for the fibroblast cells, 159 were correctly identified, but 5 were misidentified as dead PE1-E cells.

As expected, the introduction of additional variables in the heterogeneous sample affected the overall accuracy of the system. However, it was recognized that with more training and refinement, PTOLEMI’s performance could be improved to tackle more complex scenarios in clinical applications.

**Figure 5:**
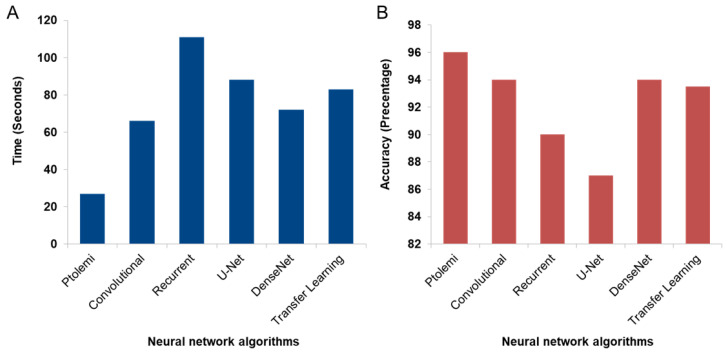
Performance Comparison of Image Analysis Algorithms. (**A**) The figure illustrates a comparison of speed among different leading image analysis algorithms. To this end, 150 images were analyzed using each of the algorithms. PTOLEMI required only 25 s to analyze the images, whereas the next fastest algorithm (CNN) took nearly three times longer. (**B**) Accuracy of the images calculated as a percentage. PTOLEMI was ~96% accurate, better than all other competing algorithms. Overall, PTOLEMI was the most accurate model and accomplished the analysis without sacrificing speed for accuracy.

Using these metrics, we can compare the accuracy and speed of PTOLEMI with other leading image analysis algorithms, such as convolutional neural network (CNN) [19], recurrent neural network (RNN) [20,21], U-Net [22], DenseNet [16,23], and transfer learning [24]. As seen in Figure 5, PTOLEMI outperforms the other algorithms in both speed (Figure 5A) and accuracy (Figure 5B), requiring only 25 s to analyze all the images, which is notably faster than the next closest algorithm-CNN, which took nearly three times longer; regarding accuracy, which was calculated as a percentage of correctly classified images, PTOLEMI presented an accuracy rate of approximately 96%, outperforming all other algorithms tested. Additionally, whereas other competing algorithms suffer from trade-off between accuracy and speed, PTOLEMI has no such problem. In personalized medicine, accuracy and speed are incredibly important, particularly in the point-of-care setting when dealing with a time-sensitive disease such as cancer without sacrificing diagnostic accuracy.

**Figure 6:**
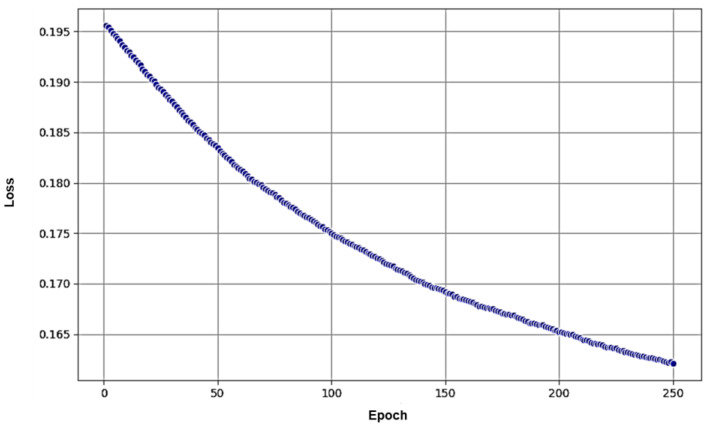
Loss over Epochs. The figure displays the variation in loss over epochs during the training of PTOLEMI’s artificial neural network. The decrease in loss signifies the model’s learning and improvement over time.

To gain further insights into the training process and model convergence, we plotted the loss over epochs during the training phase (Figure 6). The loss function served as a measure of how well the model was performing on the training data. As the training progresses, the loss should ideally decrease, indicating that the model is learning and improving its performance. From the figure, we observed a steady decrease in the loss over the course of the training, which suggested that the model was effectively learning from the data and adapting its weights and biases.

The loss function for Faster-RCNN is composed of two main parts: one for the RPN and the other for the Fast R-CNN detector. The RPN loss function involves log loss over two classes (object vs. not object) and a smooth L1 loss that robustly penalizes outliers. Similarly, the Fast R-CNN detector loss includes softmax loss over classes and bounding box regression loss. These loss functions are carefully designed to balance objectives and enhance the overall accuracy of the model [14].

To further improve accuracy, we employed several algorithms and techniques during training. The Adam optimizer, an adaptive learning rate optimization algorithm, effectively computed individual learning rates for different parameters based on estimates of first and second moments of the gradients [25,26]. By combining the advantages of AdaGrad and RMSProp, the Adam optimizer proved suitable for handling sparse gradients and online, non-stationary settings. Dropout, a regularization technique, was applied to randomly drop out units and their connections during training, preventing overfitting and acting as an ensemble learning method by averaging multiple thinned networks at test time [27]. The integration of batch normalization proved beneficial in normalizing layer inputs by adjusting and scaling activations, thereby reducing internal covariate shift during training and acting as a regularizer, enhancing the generalization of the model [28]. Lastly, data augmentation techniques were implemented to artificially increase the size and diversity of the training data by applying random transformations, such as cropping, flipping, rotating, and scaling. This augmentation process aided the model in learning invariant and robust features, effectively reducing overfitting [14].

## 4. Discussion

The current study presented PTOLEMI in combination with microfluidics. This novel integration of machine learning and artificial intelligence with a microfluidic functional assay in the field of oncology paves the way towards a point of care tool that allows efficient and effective cancer diagnosis and therapy.

While developing a point of care tool, one must consider several basic features. Accuracy and reliability, up-to-date information that allows correct decision making, a friendly and easy to use interface, high speeds for rapid patient information, offline access, cost-effectiveness, etc. PTOLEMI complementation of the microfluidic assay met all these requirements. 

The microfluidic device we described here could potentially facilitate high-throughput experimentation with minimal quantities of primary patient cell samples. It reduced labor-intensive requirements, dependence on intricate instrumentation and mitigated financial expenditures. Such microfluidic experiments may yield expedited, essential insights into potential personalized treatment options for individual patients. The integration of these advantages, coupled with the utilization of PTOLEMI image processing, enhanced analytical precision and, consequently, substantially advanced the device’s potential as an automated decision-making tool. 

PTOLEMI, Python-based Tensor Oncological Locator Examining Microfluidic Instruments, has demonstrated remarkable performance in cancer cell detection and analysis. PTOLEMI rapidly and accurately analyzed thousands of images from microfluidic devices, providing valuable insights into the sensitivities or resistances of cancer cells to different drug treatments. Based on machine learning, PTOLEMI can also learn from each new images and update its artificial neural network, enhancing its performance and adaptability. 

By enabling analysis at the point of care and within an expedient time scale, PTOLEMI overcomes bottlenecks commonly encountered in centralized lab systems, addressing the critical issue of time sensitivity in personalized cancer treatment. 

One notable challenge in the development processes of AI-based tools is the requirement for substantial amounts of data needed for the training of the model, increasing its efficiency. As more diverse and comprehensive datasets become available, PTOLEMI can enhance its ability to accurately identify and classify different cell types within biopsy samples, increasing the specificity of personalized cancer treatment plans.

Machine-learning algorithms in the image analysis field can incorporate new insights and nuances that human capabilities will never have, and that may reveal important information that may progress cancer treatment. Moreover, future steps for PTOLEMI involve expanding its capabilities to encompass a broader range of cancer-related analyses. This includes the detection and analysis of various biomarkers, genetic mutations, and molecular characteristics associated with different cancer types. Adding such aspects into PTOLEMI analysis can provide a more comprehensive assessment of tumor heterogeneity and guide personalized treatment decisions with even greater precision.

As demonstrated, PTOLEMI has immense potential as a user friendly, point of care diagnostic tool. The combination of PTOLEMI with a microfluidic device can set a new standard in oncological decision assistance at the point of care. With the image analysis being cut down from 1–3 weeks to less than 20 min, a given patient can be biopsied and begin a personalized treatment regimen in less than three days, a currently unheard of timeframe. 

Data management, accessibility, security, and the efficient use of storage resources are some of the challenges that science is facing today. On-spot image analysis achieved by PTOLEMI eliminates the need for large memory storage capacities and enables off-line analysis that is critical for point of care applications.

The integration of AI-driven platforms like PTOLEMI in the field of medicine aligns with the growing trend in leveraging advanced technologies to revolutionize healthcare practices. Embracing AI-driven platforms in personalized cancer treatment is timely and crucial. By adopting AI technology, we can harness its power to save lives and expedite progress in cancer treatment. This new combined platform is not only more effective, precise, and may increase survival rates amongst patients, but it also conserves financial resources, which may be addressed for the benefits of health care services.

## Data Availability

The datasets used and analyzed during the current study are available from the corresponding author on reasonable request.

## Supplementary Materials

The following supporting information can be downloaded at: https://www.mdpi.com/article/10.3390/diagnostics13193075/s1, Figure S1: Cell culture chip size comparison. A cell culture chip with red dye for better contrast next to a New Israeli Shekel coin (diameter 18mm) for scale. Figure S2: Culture Media Diffusion Simulation- Cells were seeded in the chip and isolated in respective culture chambers. Red dye, meant to simulate culture media was inserted into the channels, with the neck valve closed such that the medium can only diffuse (reddish discoloration and the bottom of each chamber) in and out of the culture chambers rather than flow directly into them, thus better simulating in vivo conditions. Figure S3. Neuron arrangement and Interactions with a single chamber Image (size: 512 × 512 px). Arrangement and interactions of neurons in PTOLEMI’s artificial neural network (ANN) for image analysis. The ANN comprises multiple layers of interconnected neurons, allowing for intricate data processing and feature extraction from cell images. The weights and biases assigned to each neuron govern the transmission of data between layers. Figure S4: Analysis of Glioma cells- comparative analysis between human assessments and PTOLEMI’s AI model. (A) Confusion Matrix for PTOLEMI’s Classification Results on a sample of 150 Images: The confusion matrix shows True Positive (TP), False Negative (FN), False Positive (FP), and True Negative (TN) values, indicating PTOLEMI’s performance on the image dataset. (B) Visual representation of image identification: Glioma 2490 stem cells cells were seeded in the microfluidic device and stained with Propidium Iodide (magenta, dead cells) and Hoechsht 33342 (Blue, live). Image analysis was then conducted using PTOLEMI.
