# Supplementary material for: PTOLEMI: Personalized Cancer Treatment through Machine Learning-Enabled Image Analysis of Microfluidic Assays
Source: Diagnostics (Basel). 2023 Sep 28;13(19):3075. doi: 10.3390/diagnostics13193075 (PMC10572730; doi:10.3390/diagnostics13193075)
Supplement: Supplementary file 1 [file diagnostics-13-03075-s001.zip › diagnostics-2575201-supplementary.pdf]

Supplementary Information

PTOLEMI: Personalized Cancer Treatment through Machine Learning-Enabled Image Analysis of Microfluidic Assays

Bernard Moerdler, Matan Krasner, Elazar Orenbuch, Avi Grad, Benjamin Friedman, Eliezer Graber, Efrat Barbiro-Michaely and Doron Gerber*

Life Sciences Faculty and Nanotechnology Institute, Bar-Ilan University, Ramat Gan 5290002, Israel
* Correspondence: doron.gerber@biu.ac.il

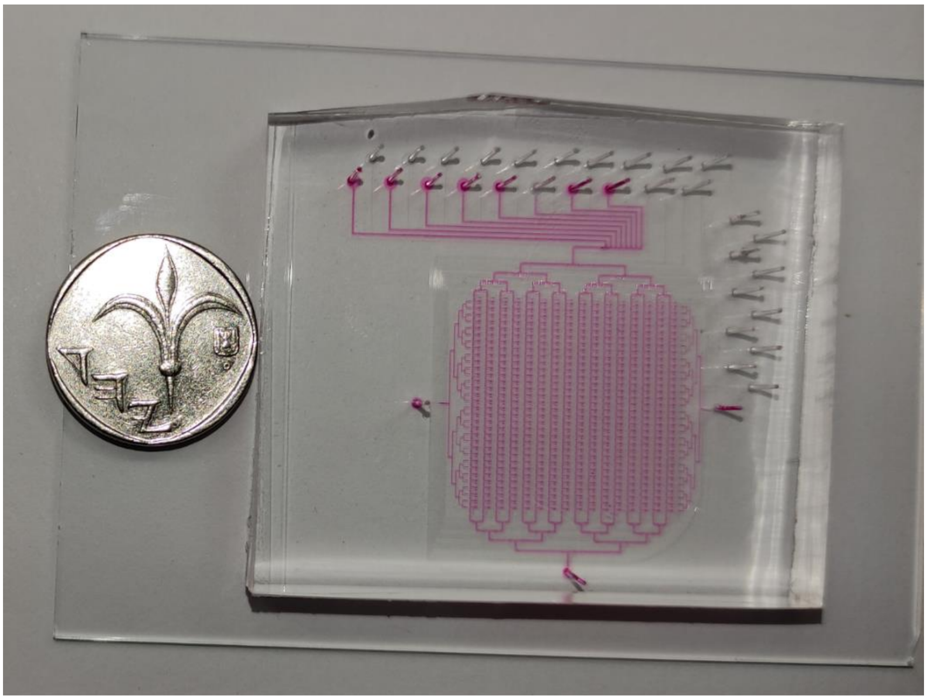

Supplementary Figure S1: Cell culture chip size comparison. A cell culture chip with red dye for better contrast next to a New Israeli Shekel coin (diameter 18mm) for scale.

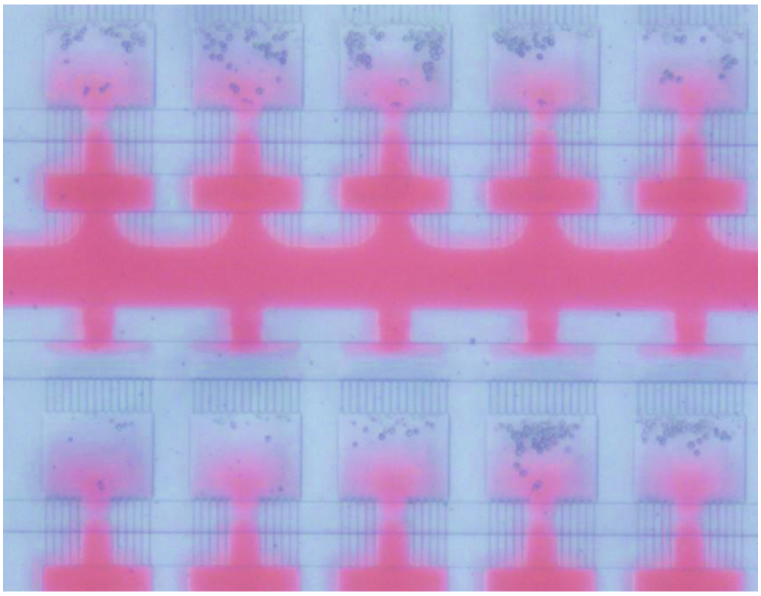

Supplementary Figure S2: Culture Media Diffusion Simulation- Cells were seeded in the chip and isolated in respective culture chambers. Red dye, meant to simulate culture media was inserted into the channels, with the neck valve closed such that the medium can only diffuse (reddish discoloration and the bottom of each chamber) in and out of the culture chambers rather than flow directly into them, thus better simulating *in vivo* conditions.

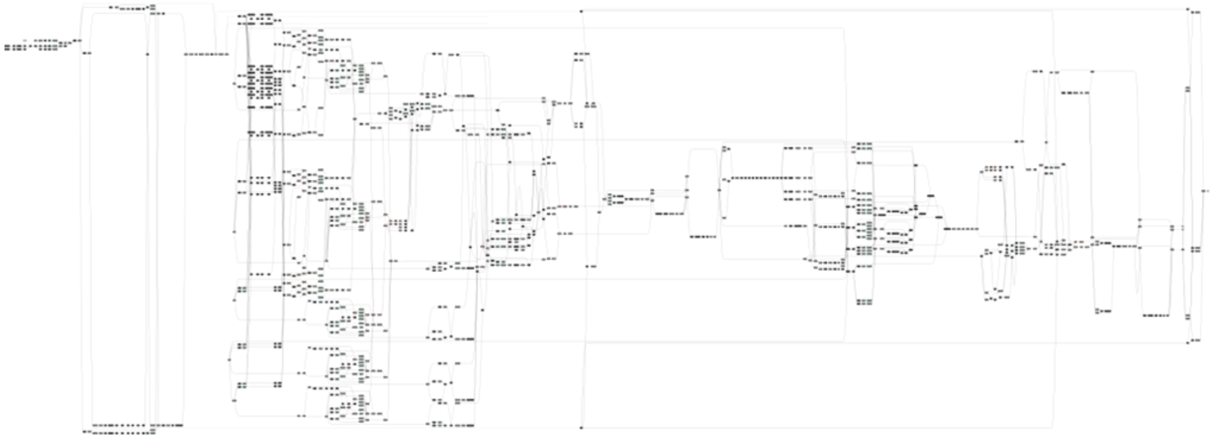

Supplementary Figure S3. Neuron arrangement and Interactions with a single chamber Image (size: 512x512px). Arrangement and interactions of neurons in PTOLEMI's artificial neural network (ANN) for image analysis. The ANN comprises multiple layers of interconnected neurons, allowing for intricate data processing and feature extraction from cell images. The weights and biases assigned to each neuron govern the transmission of data between layers.

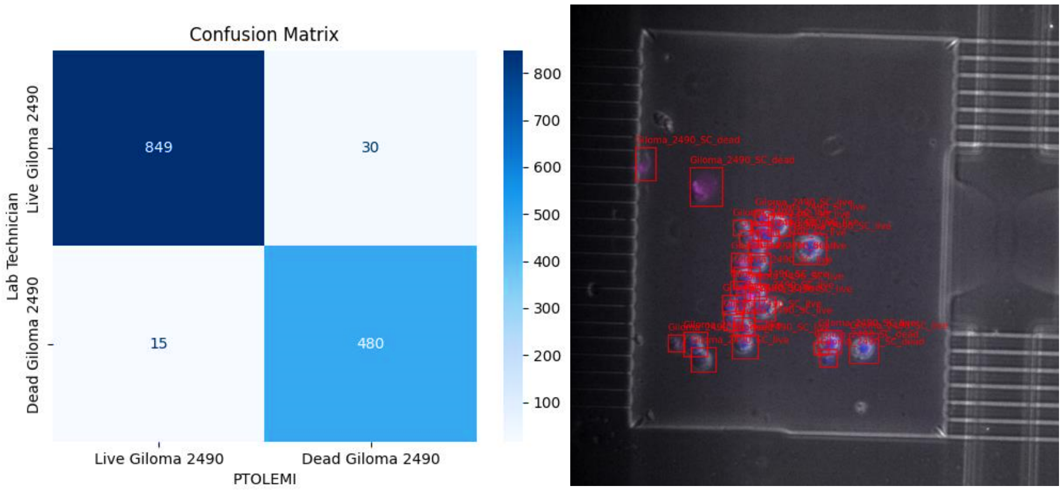

Supplementary Figure S4: Analysis of Glioma cells- comparative analysis between human assessments and PTOLEMI's AI model. A) Confusion Matrix for PTOLEMI's Classification Results on a sample of 150 Images: The confusion matrix shows True Positive (TP), False Negative (FN), False Positive (FP), and True Negative (TN) values, indicating PTOLEMI's performance on the image dataset. B) Visual representation of image identification: Glioma 2490 stem cells cells were seeded in the microfluidic device and stained with Propidium Iodide (magenta, dead cells) and Hoechsht 33342 (Blue, live). Image analysis was then conducted using PTOLEMI.
